# Dynamic Changes of Endogenic or Exogenic β-Carboline Alkaloid Harmine in Different Mammals and Human *in vivo* at Developmental and Physiological States

**DOI:** 10.3389/fnagi.2021.773638

**Published:** 2022-01-14

**Authors:** Ning Cao, Shuping Li, Aimin Xu, Manlin Li, Xiaoguang Zou, Zunji Ke, Gang Deng, Xuemei Cheng, Changhong Wang

**Affiliations:** ^1^The MOE Key Laboratory for Standardization of Chinese Medicines, Shanghai Key Laboratory of Chinese Compound Medicines, Institute of Chinese Materia Medica, Shanghai University of Traditional Chinese Medicine, Shanghai, China; ^2^Kashi Prefecture First People’s Hospital, Kashi, China; ^3^School of Basic Medicine, Shanghai University of Chinese Medicine, Shanghai, China

**Keywords:** harmine, β-carboline alkaloids, exposure levels, endogenous substance, Alzheimer’s disease

## Abstract

**Objective:**

Several β-carboline alkaloids (βCBs), such as harmine, harmaline, harmane, and nor-harmane, are effective for Alzheimer’s disease mouse models. They can be found in some plants, common foodstuffs, and blank plasma of various mammals. However, whether these compounds in mammals are exogenous or endogenous remain unclear.

**Methods:**

The exposure levels of βCBs and of neurotransmitters in plasma and tissues of pup rats, aging rats, mice of different physiological states, and healthy volunteers were detected by using UPLC-MS/MS. Plasma and tissue samples from 110 newborn rats up to 29 days old at 11 sampling points were collected and were analyzed to determine the concentration variation of βCBs in the developmental phase of newborn rats. The plasma of rats aged 2 to 18 months was used to detect the variation trend of βCBs and with some neurotransmitters. The plasma samples of normal C57BL/6 mice, APP/PS1 double transgenic mice, and scopolamine-induced memory impairment mice were collected and were analyzed to compare the difference of βCBs in different physiological states. The exposure levels of βCBs such as harmine, harmaline, and harmane in plasma of 550 healthy volunteers were also detected and analyzed on the basis of gender, race, and age.

**Results:**

Results showed that harmine was the main compound found in rats, mice, and human, which can be detected in a newborn rat plasma (0.16 ± 0.03 ng/ml) and brain (0.33 ± 0.14 ng/g) without any exogenous consumption. The concentration of harmine in rat plasma showed a decreasing trend similar to the exposure levels of neurotransmitters such as 5-hydroxytryptamine, acetylcholine chloride, glutamic acid, tyrosine, and phenylalanine during the growth period of 18 months. The harmine exposure in rats and human indicates high dependence on the physiological and pathological status such as aging, gender, and race.

**Conclusion:**

The dynamic changes of harmine exposure in different animals and human, *in vivo*, at developmental and physiological states indicate that harmine is a naturally and widely distributed endogenous substance in different mammals and human. In addition to exogenous ingestion, spontaneous synthesis might be another important source of harmine in mammals, which should be verified by further experiment.

## Introduction

The β-carboline alkaloids (βCBs), such as harmine, harmaline, harmane, and nor-harmane, are active components of *Peganum harmala* (Li et al., 2017a). Traditionally, *P. harmala* is used to treat diseases, such as cough, asthma, rheumatoid arthritis, and swelling pain in regions such as the Middle East, central Asia, and South America (Zhao et al., 2012). In addition, such βCBs show various pharmacological effects, including hypoglycemic effect, antineoplastic activity, and acetylcholinesterase (AChE) inhibitory activity (Li et al., 2017a). These βCBs are also present in other plants, including *Banisteriopsis caapi*, *Tribulus terrestris*, and *Ayahuasca* (Li et al., 2016). Such βCBs can also be found in common foodstuffs, such as plant-derived foods (e.g., grapes, rice, corn, barely, bean, and rye), processed foods (e.g., wine, beer, whiskey, brandy, sake, coffee, vinegar, and tobacco), and meat products (e.g., barbecue, smoked fish, and smoked sausages) (Xie et al., 2021). Moreover, both harmane and nor-harmane are widely distributed in the brain, liver, blood, and urine of human and many other mammals (Louis et al., 2005, 2010, 2013). Based on previous reports, plasma contents of harmane in patients with tremor and Parkinson’s disease were higher than those in healthy individuals (Louis et al., 2005, 2014). Harmine, harmaline, and other βCBs have been found in the blank plasma of adult rats and mice even without consuming such compounds (Li et al., 2016). Consequently, whether harmine, harmaline, harmane, and nor-harmane are endogenous or exogenous in mammalian tissues and plasma, and whether such βCBs in mammals are useless or functional, remain unclear.

The Pictet–Spengler reaction (P–S reaction) in plants synthesized the βCBs (Cao and Wang, 2021). Strictosidine synthase is a vital enzyme in the reaction (Stockigt et al., 2011). In addition, a protein similar to strictosidine synthase is found in the human brain, which may indicate that the P–S reaction may also occur in human (Fabbri et al., 2000). Tryptamine is a synthetic precursor of the P–S reaction. Moreover, tryptamine is widely distributed in the brain (Abu Ghazaleh et al., 2015). Thus, harmaline, harmine, harmane, and nor-harmane may be self-synthesized in mammals. Furthermore, the function of such βCBs in mammals remains unknown.

Based on previous reports, βCBs can play various pharmacological effects, such as anticoagulant activity, hypoglycemic effect, antineoplastic activity, antioxidative, and anti-inflammatory activity (Li et al., 2017a). These βCBs are either AChE and butyrylcholinesterase inhibitors or monoamine oxidase-A (MAO-A) inhibitors (Li et al., 2017a). In addition, the four abovementioned alkaloids can bind with opioid receptors, imidazoline I_2_ receptors, and 5-hydroxytryptamine (5-HT) receptors, which contribute to the analgesic effect, reduction of withdrawal syndrome, and regulation of neurotransmitters (Li et al., 2017a). Moreover, neurotoxicity of harmine, harmaline, harmane, and nor-harmane is an indivisible side effect, which leads to essential tremor (Wang et al., 2019).

Based on our published research and results, harmine, harmaline, harmane, and nor-harmane are effective in mice with nervous system diseases, such as Alzheimer’s disease (AD) (Li et al., 2018). AD is a common neurodegenerative disease, which is characterized by a decline in memory, language, and other cognitive skills (Association, 2016). Although no conclusion has been found on the pathogenesis of AD, several recognized hypotheses have been carried out. A popular mechanism is self-replication and spreading of the Aβ and Tau aggregates (Kumar et al., 2015). Not labeled by thioflavin or Congo red-based probes, Aβ deposition in transgenic mice is similar to brain tissue of patients with AD (Hampel et al., 2018). In addition, the cholinergic system of the brain plays an important role in AD. Cholinesterase inhibitors can increase the availability of acetylcholine ACh at synapses in the brain (Hampel et al., 2018). The effective cholinesterase inhibitors such as donepezil, galantamine, and rivastigmine have been proven to be clinically useful in AD treatment (Perng et al., 2018). Moreover, neurotransmitters play an indispensable role in AD development, and aberrant neurotransmitter release at synapses can cause cognitive decline in AD (Tang, 2019). Researchers have found that AD is associated with inadequate levels of various neurotransmitters (Kumar et al., 2015). The AChE inhibitory activity and regulation of neurotransmitters are the potential underlying mechanisms of harmine, harmaline, harmane, and nor-harmane in the treatment of AD mouse models. Thus, such alkaloids, which are widely distributed in the body fluids of the mammals, may improve the prevention and treatment of AD.

Therefore, harmaline, harmine, harmane, and nor-harmane may be endogenous substances, and a potential relationship can be found between such endogenous alkaloids and AD.

The plasma of rats, mice, and human at different ages were tested, particularly the newborn pup rats, to verify whether harmaline, harmine, harmane, and nor-harmane are endogenous and naturally present in mammals. The AD is a cognitive impairment disease, which is closely associated with age. Brain development in childhood, particularly born within a month, is crucial to cognitive function (Mollon et al., 2018). Memory and learning ability have developed from infancy to adulthood based on processing speed, working memory, language, and visuospatial test (Mollon et al., 2018). However, cognition decreases with age (Guerreiro and Bras, 2015). According to research published in 2016, most people with AD are aged 65 or older, and 15% of people with AD are aged 65–74, whereas 44% are aged 75–84 (Association, 2016). Growth and aging are important processes of cognitive level development. Thus, the contents of harmine, harmaline, harmane, and nor-harmane in different developmental states of mammals are determined. In this article, the developmental stage of rats is defined as less than 29 days old, whereas aging is defined as between 2 and 18 months of age.

Plasma of AD mouse models is tested and compared with those of the control animals’ in accordance with the possible mechanism of AD to confirm whether the content variation of harmine, harmaline, harmane, and nor-harmane in mammal plasma implies the occurrence of AD. Exposure levels of these βCBs in healthy volunteers were also detected to explore the relationship between βCBs exposure of patterns and age, as well as of gender and race.

In general, the abovementioned research is important to determine the origin and functions of harmine, harmaline, harmane, and nor-harmane in mammal plasma. Studies on the relationship among plasma contents of such βCBs in different developmental and physiological states of mammals can provide a comprehensive understanding of the endogenous physiological effects of such alkaloids. Furthermore, the results may provide insights into the anti-AD functional foods and drugs.

## Materials and Methods

### Reagents and Materials

Harmine, harmane, and harmaline (purity > 98%) were isolated by HPLC from the seeds of *P. harmala* in our laboratory. Nor-harmane was purchased from Sigma Aldrich Co. (St. Louis, MO, United States). Scopolamine hydrobromide was purchased from TCI (Shanghai) Development, Co., Ltd. (Shanghai, China). The _L_-tryptophan (_L_-Trp), 5-hydroxytryptamine (5-HT), 5-hydroxyindole-3-acetic acid (5-HIAA), acetylcholine chloride (ACh), choline chloride (Ch), _L_-glutamic acid monosodium salt monohydrate (_L_-Glu), _L_-phenylalanine (_L_-Phe), _L_-tyrosine (_L_-Tyr), theophylline, tacrine (internal standard), and heparin sodium were purchased from Sigma Aldrich Co. (St. Louis, MO, United States). Perchloric acid and sodium hydroxide were purchased from Meilunbio^®^ Biotech, Co., Ltd. (Dalian, China). Bovine serum albumin was purchased from YEASEN Biotechnology, Co., Ltd. (Shanghai, China). The HPLC-grade acetonitrile, methanol, and formic acid were purchased from Fisher Scientific, Co. (Santa Clara, CA, United States). Deionized water (>18 mΩ) was purified by Milli-Q Academic System (Millipore, Corp, Billerica, MA, United States).

### Animals

Twenty adult and 11 pregnant Sprague-Dawley rats, and 10 C75BL/6 mice were obtained from the Drug Safety Evaluation and Research Center of Shanghai University of Traditional Chinese Medicine. Ten APP/PS1 double transgenic mice in C57BL/6 background aged 5–6 months with their age-matched littermates were obtained from Nanjing Biomedical Research Institute of Nanjing University (Nanjing, China). Animals were housed in a well-lighted air-conditioned room under standard environmental conditions (room temperature and relative humidity were kept at 25°C ± 1°C and 60–65%, respectively) and given free access to rodent chow and tap water prior to the study. All procedures using animals were in accordance with the regulations for animal experimentation issued by the State Committee of Science and Technology of the People’s Republic of China on 14 November 1988 and approved by the Animal Ethics Committee of Shanghai University of Traditional Chinese Medicine (NO. PZSHUTCM190912018; Approval date: 12 September, 2019).

### Voluntary Subjects

Participants were recruited from two cohorts: Shanghai University of Traditional Chinese Medicine (i.e., the SH cohort) and Kashi Prefecture First People’s Hospital, Xinjiang, China (i.e., the XJ cohort). This study focused on the exposure rules of harmine, harmaline, harmane, and nor-harmane in plasma of non-AD patients with different ages, genders, and races. Therefore, all participants were normal individuals, with a low likelihood of clinical AD. During recruitment, cognitive function can be assessed using a variety of simple, informal techniques in accordance with the National Institute on Aging-Alzheimer’s Association Guidelines (Albert et al., 2011). A total of 131 participants were recruited from the SH cohort. Most of them were students aged 24 to 30, and 15 volunteers were staffs aged 31 to 55. The other 419 participants were enrolled for the XJ cohort. All of them went to a hospital for physical examination. The 419 healthy volunteers were in the age range of 19–81 years old. All subjects were given an informed consent and asked no intervention before and throughout the study period. Exclusion criteria were not available. The study was approved by the Research Ethics Committee of Kashi Prefecture First People’s Hospital, and all participants provided an informed consent (NO. 2017-03; Approval date: February 28, 2017).

### Analysis of Target βCBs in Plasma and Tissues of Mammals by UPLC-ESI-MS/MS

The concentrations of alkaloids were quantified using the SHIMADZU LC-30AD UPLC system (Shimadzu, Kyoto, Japan) connected to an AB Sciex QTRAP^®^ 6500 triple quadrupole mass spectrometer (SCIEX, United States) equipped with an ESI source using positive ion detection mode for multiple reaction monitoring. Based on a previously validated method, chromatographic separation was conducted using a UPLC BEH C_18_ column (50 mm × 2.1 mm, 1.7 μm, Waters, United States) (Zhao et al., 2011). Given the difference in the linearity range, the sample pre-treatment was slightly adjusted, and the methods and results are shown in Supplementary Figure 1 and Supplementary Tables 1–3, respectively. The mobile phase consisted of an aqueous solution of 0.1% formic acid (solvent A) and acetonitrile (solvent B) with a flow rate of 0.4 ml/min. The gradient elution was established as follows: 0–2.5 min, 9–13% B; 2.51–3 min, 14–14.5% B; 3–4 min, 14.5–15.5% B; 4–5 min, 15.5–90% B; 5–6 min, 90–90% B; 6–7 min, 90–9% B; 7–8 min, 9% B. All other instrumental parameters were set in accordance with previous studies, and the method was well validated and successfully applied to determine the concentrations of alkaloids (Zhao et al., 2011).

### Analysis of Neurotransmitters in Plasma of Mammals by UPLC-ESI-MS/MS

The concentrations of neurotransmitters, including _L_-Trp, 5-HT, 5-HIAA, ACh, Ch, _L_-Glu, _L_-Phe, and _L_-Tyr, were determined using a SHIMADZU LC-30AD UPLC system (Shimadzu, Kyoto, Japan) connected to an AB Sciex QTRAP^®^ 6500 triple quadrupole mass spectrometer (SCIEX, United States) equipped with an ESI source. Chromatographic separation was conducted using a ZIC-cHILIC column (150 mm × 2.1 mm, 3 μm) with a SeQuant ZIC-cHILIC guard column (20 mm × 2.1 mm, 5 μm, Merck-Sequant, Germany). The mobile phase consisted of an acetonitrile (A)–water mixture containing 0.1% formic acid (B), and the gradient elution was established as: 0–8 min, 65% A. All other instrumental parameters and sample pre-treatment were set in accordance with previous studies, and the method was well validated and successfully applied to determine the concentrations of neurotransmitters (Jiang et al., 2019).

### Exposure Levels of Target Alkaloids in the Developmental Phase of Newborn Rats Within 29 Days

**FIGURE 1:**
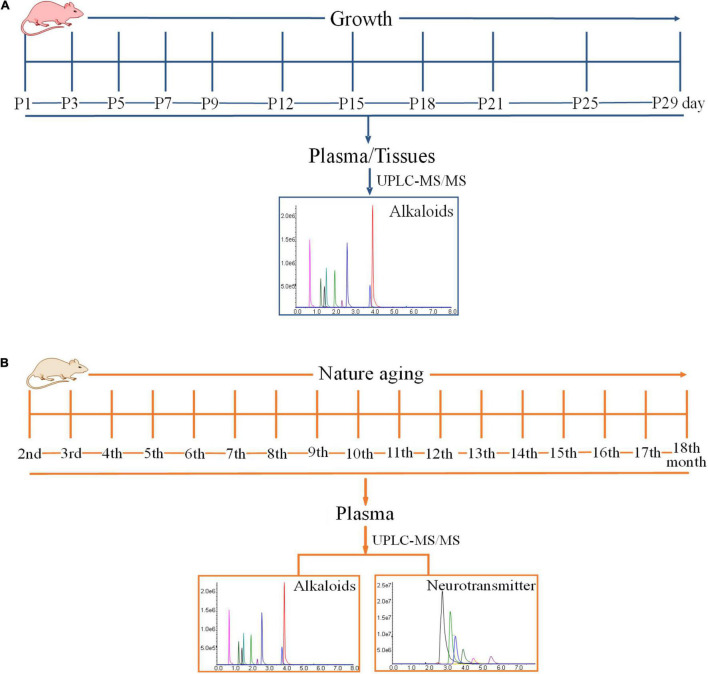
Time schedules of experiment. **(A)** The time schedule of the experimentation on the exposure levels of alkaloids in the developmental phase of newborn rats within 29 days of birth. **(B)** The time schedule of the experimentation on the exposure levels of alkaloids and neurotransmitters during the growth of 18-month-old rats (from youth to old stage).

Eleven Sprague-Dawley pregnant rats were used in this study. The pregnant rats were raised under an environmentally controlled room with free access to food and water all throughout the experiment. After parturition, pup rats were grouped into 11 on the basis of mother rats. The individual group indicated one sampling point and contained 10 pup rats obtained from 10 different mother rats. The pup rats were sacrificed at postnatal day 1 (P1), 3 (P3), 5 (P5), 7 (P7), 9 (P9), 12 (P12), 15 (P15), 18 (P18), 21 (P21), 25 (P25), and 29 (P29) for sample collection, in which 11 sampling points were collected. The specific time is shown in Figure 1A. Each pregnant rat could give birth to around 10 pup rats, and every pup rat was sacrificed at certain time to ensure that each point in time contained 10 samples (five female and five male). The sex of pup rats was determined based on the anogenital distance. Pup rats were anesthetized at specified time for sample collection, and the grouping and sampling time is shown in Supplementary Table 4. Approximately 0.5 ml of blood sample was collected from the angular vein of each rat and transferred into a 1.5 ml heparinized tube. The supernatant plasma (100 μl) was transferred into another 1.5 mL centrifuge tube after the centrifugation of blood at 3,000 × *g* and 4°C for 10 min. The tissues of the brain, heart, liver, spleen, lung, kidney, genital organ, muscle, white fat, and brown fat of each pup rat were also collected. Various tissues were weighted and stored at a suitable tube. All plasma and tissue samples were stored at −80°C until analysis.

### Exposure Levels of Alkaloids in Fodder and Bedding

The concentrations of harmine, harmaline, harmane, and nor-harmane in the fodder and bedding were detected. The fodder and bedding were obtained from the cages of pregnant rats and their pup rats. The detection method was referred to a quality specification established by Yang et al. (2014). The fodder and bedding were accurately powdered and weighted. The powders were added to methanol, which was 25 times its volume, and were ultrasound-treated for 25 min. In addition, power and frequency were kept at 250 W and 30 kHz, respectively. Afterward, supernatant (5 mL) was transferred to another tube and evaporated to dry at 37°C under a slight stream of nitrogen. The dried residue was reconstituted with 100 μl of 9% acetonitrile and vortexed for 2 min. After centrifugation at 13,000 × *g* and 4°C for 10 min, 20 μl of supernatant was injected into the UPLC-ESI-MS/MS system for alkaloid analysis.

### Exposure Levels of Alkaloids and Neurotransmitters During the Growth of 18-Month-Old Rats (From Youth to Old Stage)

Twenty Sprague-Dawley rats (200–220 g), with 10 males and 10 females, were used in this study. The rats were raised under an environmentally controlled room with free access to food and water all throughout the experiment. The experimentation lasted for 16 months. Blood samples were collected once a month, and blood was drawn at around 9:00 am to 11:00 am. The schematic diagram is shown in Figure 1B. Blood samples were collected from the angular vein for hematological analyses every month after rats were anesthetized with isoflurane. The blood samples were promptly centrifuged at 3,000 × *g* and 4°C for 10 min, and all of the supernatant plasma was transferred into a new 1.5 ml centrifuge tube. Plasma samples were stored at −80°C until analysis. After sample collection, plasma was used to analyze some alkaloids and neurotransmitters after pretreatment.

### Alkaloid Exposure Levels in Different Physiological States of Mice

Two AD mouse models were used, including 10 male APP/PS1 double transgenic mice and 10 male scopolamine molding mice. All mice were housed under an environmentally controlled room with free access to food and water before the experiment. Based on the results of the Morris Water Maze test, the APP/PS1 double transgenic mice had an impaired spatial learning and memory compared with C57BL/6 mice (He et al., 2015). Mice were anesthetized with isoflurane, and blood was collected from the right orbital vein (He et al., 2015). The blood samples were centrifuged at 3,000 × *g* and 4°C for 10 min. Then, supernatant plasma was collected and stored at −80°C until analysis. Another AD mouse model was composed of male C57BL/6 mice molded through intraperitoneal injection of scopolamine (1 mg/kg) for 7 days (Li et al., 2018). Compared with C57BL/6, the scopolamine-molded mice had an impaired memory based on the results of the Morris Water Maze test (Li et al., 2018). After molding, the model mice and control were anesthetized with isoflurane, and blood was collected from the right orbital vein. The blood samples were centrifuged at 3,000 × *g* and 4°C for 10 min. Then, supernatant plasma was collected and stored at −80°C until analysis.

### Exposure Levels of Alkaloids in Different Developmental and Health Conditions in Human

All 550 individuals were given written informed consents before blood was drawn. In addition, the participants and their relatives were informed that the anonymized data might be used in clinical research studies. Moreover, health examination and questionnaires were necessary. The guardian was requested to answer the questionnaires if participant could not answer. The survey involved various aspects, including name, age, nationality, gender, living habits, and contact information. Living habits included drinking and smoking. Detail information is shown in Table 1. Health examination about physiological states included neurodegeneration and any other disease. This experiment aimed to detect exposure levels of harmine, harmaline, harmane, and nor-harmane in various developmental and physiological states. Furthermore, the difference of alkaloid exposure levels by gender, race, and lifestyle was detected. Blood sampling and numbering were directed by nurses in Kashi Prefecture First People’s Hospital and Shuguang Hospital affiliated to Shanghai University of Traditional Chinese Medicine. Serial numbers corresponded to the detailed information about every participant. The numbers were randomly allocated by sequence of arrival at hospital. Analysis about alkaloids in plasma was developed without further information except for serial numbers. Approximately 1 ml of venous blood was collected from participants who have fasted for at least 12 h. The blood samples were centrifuged at 3,000 × *g* and 4°C for 10 min to obtain plasma. Then, plasma samples were stored at −80°C until analysis.

**TABLE 1:** Characteristics of healthy participants.

Characteristics of subjects	SH cohort	XJ cohort
Number	131	419
Gender (women/men)	79/52	101/318
Age (mean ± SD)	28 ± 4	41 ± 12
Race (Han/Uyghur)	124/7	274/145
Drinking (yes/no)	13/118	199/220
Smoking (yes/no)	9/122	162/257

*-no relevant statistics.*

### Statistical Analysis

Statistical evaluation was performed using SPPS version 18, and the data were presented as mean ± SD. Unadjusted *P* values were reported as this study used an exploratory rather than confirmatory analysis. This study aimed to confirm whether harmine, harmaline, harmane, and nor-harmane were endogenous and compare their exposure levels in different gender, nationality, and lifestyle. Thus, four separate stepwise logistic regression analyses were conducted, which is one analysis each for harmine, harmaline, harmane, and nor-harmane, serving as the dependent variable. Data distribution was graphically evaluated using histograms and Q–Q plots. Non-parametric and parametric tests were used in the study. The *T* test was used to compare the concentrations of alkaloids among the different groups. A non-parametric test was applied as part of the statistical analyses for non-normal distribution. Participants were divided into two groups, and 60 years old was selected as the division point. Kruskal–Wallis H non-parametric test was used to compare the two groups. The threshold for statistical significance was set at *P* < 0.05 (2-tailed).

## Results

### Alkaloid Exposure Levels in the Developmental Phase of Newborn Rats Within 29 Days After Birth

**FIGURE 2:**
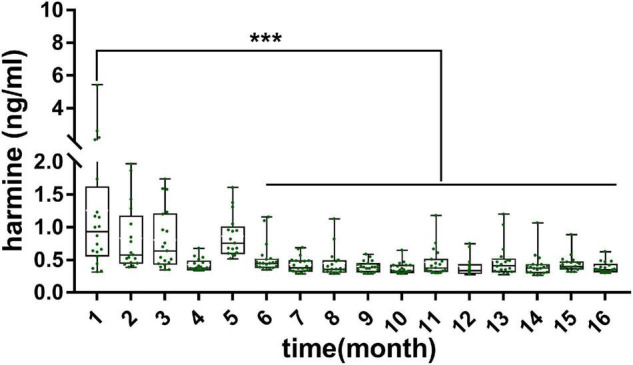
Concentration of harmine in rat plasma and various tissues during the development of pup rats. Significant difference: ***P < 0.001.

The validated analytical method was applied to study the exposure levels of alkaloids in plasma and various tissues with the growth of pup rats within a month. The plasma concentrations of harmine were calculated by using the calibration curve. However, the concentrations of harmaline, harmane, and nor-harmane were difficult to count when the contents were below the lower limit of detection. As shown in Figure 2, the contents of harmine in plasma and in other organs showed different variations. First, harmine was detected in plasma and all tissues. The contents were listed as follows: plasma, 0.16 ± 0.03 ng/ml; brain, 0.33 ± 0.14 ng/g; heart, 0.34 ± 0.15 ng/g; liver, 0.26 ± 0.11 ng/g; spleen, 0.37 ± 0.12 ng/g; lungs, 0.46 ± 0.11 ng/g; kidney, 0.44 ± 0.13 ng/g; genital organ, 0.39 ± 0.12 ng/g; white fat, 0.33 ± 0.07 ng/g; brown fat, 0.36 ± 0.17 ng/g; and muscle, 0.31 ± 0.18 ng/g. The concentrations of harmine remarkably changed with time because of the various developmental characteristics of different organs. The concentrations of harmine in the plasma, brain, white fat, and genital organ decreased, whereas other organs (heart, liver, spleen, lungs, kidney, muscle, and brown fat) showed no significant trend with the growth of pup rats. Plasma content stabilized on the ninth day (*P* > 0.05), whereas the concentrations in the brain and white fat stabilized on the fifteenth and twenty-first day, respectively (*P* > 0.05). The contents of harmine in plasma, brain, and white fat of 3-day-old rats were significantly lower than those of 1-day-old rats but higher than those at the plateau stage (*P* < 0.05). No plateau was observed in the content of harmine in the genital organ, and it was decreasing everyday (*P* < 0.001). The brain was a crucial functional organ of harmine, whereas the liver was a vital metabolic organ. Then, the amounts of harmine in various tissues were calculated, and the amount–time curves were drawn (Supplementary Figure 2). The absolute amount of harmine increased in tissues of the brain, heart, liver, spleen, lungs, and kidney with the growth of pup rats. In tissues of the heart, lungs, and kidney, the absolute amount of harmine increased with the development of organs until the third week after birth. Notably, the absolute amount of harmine in the brain increased faster than the development of body weight in the second week after birth.

Harmine, harmaline, harmane, and nor-harmane were not detected in fodder and bedding. A weak signal response was detected at the same peak time in harmine, and all of the responses were below the lower limit of quantification after the supernatant was concentrated 50 times. The detail dates are shown in the Supplementary Table 5.

### Exposure Levels of Alkaloids and Neurotransmitters During the Growth of 18-Month-Old Rats (From Youth to Old Stage)

**FIGURE 3:**
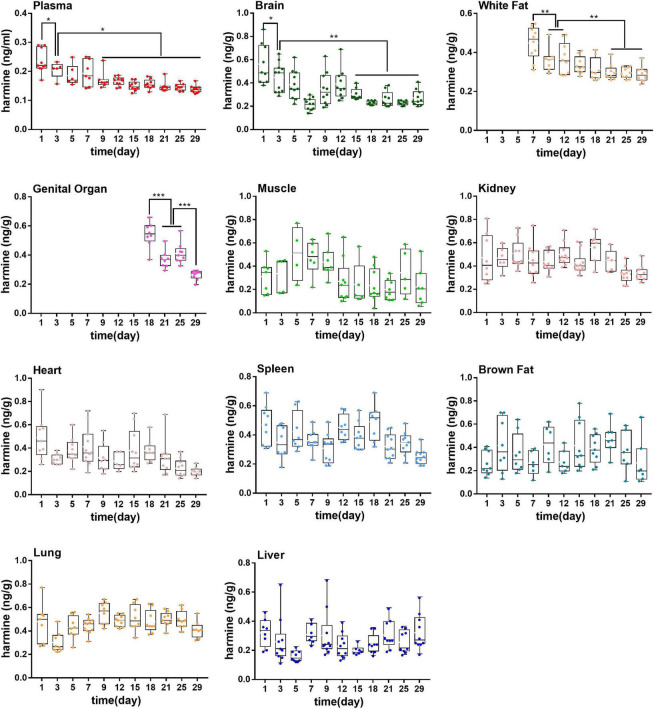
Concentrations of harmine in rat plasma during the growth of 18-month-old rats (from youth to old stage). *P < 0.05, **P < 0.01, ***P < 0.001.

The alkaloids in plasma during the aging of rats were determined by using a validated analytical method. As shown in Figure 3, the concentrations of harmine in plasma decreased gradually with aging. The contents of harmine reached 1.80 ± 1.51 ng/mL in the first month and reduced to 0.35 ± 0.04 ng/ml in the sixteenth month. The concentrations of harmine in the first month were significantly higher than those in the sixth to sixteenth month (*P* < 0.01). Moreover, in the second to fifth month, the contents of harmine showed a decreasing trend with slight fluctuation. The concentrations stabilized in the sixth month and maintained at a low concentration (*P* > 0.05).

**FIGURE 4:**
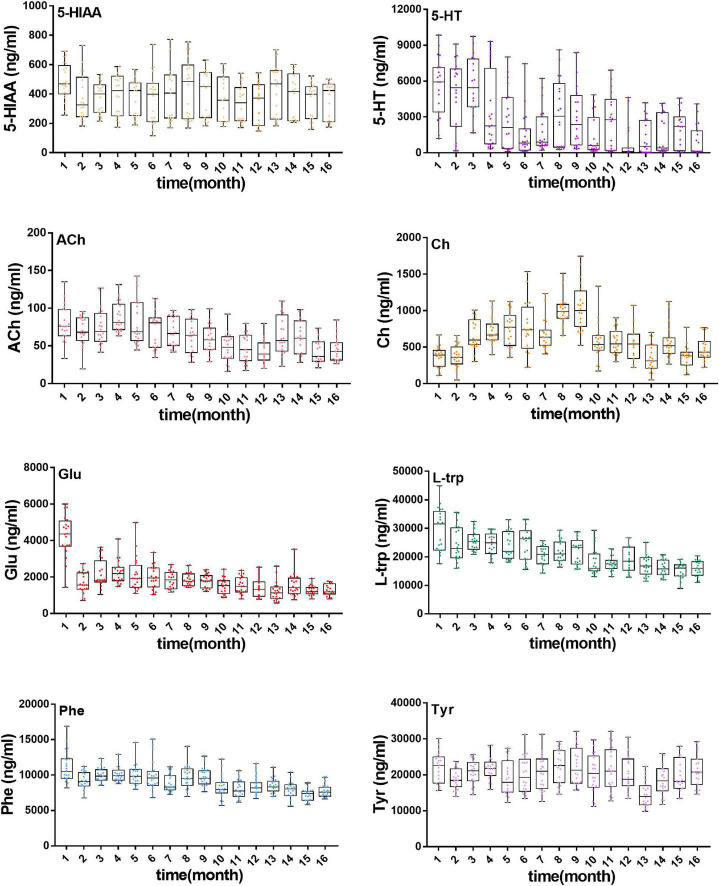
Concentration of eight neurotransmitters in rat plasma during the growth of 18-month-old rats (from youth to old stage), including 5-HIAA, 5-HT, ACh, Ch, Glu, L-Trp, Phe, Tyr.

Meanwhile, neurotransmitter concentration in plasma during aging of rats was determined by using a validated analytical method. As shown in Figure 4, various neurotransmitters showed different trends (concentration range, mean ± SD): 5-HIAA (154.7–691, 384.3 ± 151.3 ng/ml), 5-HT (45.4–9850.2, 2,653 ± 2,644 ng/ml), ACh (30.1–137.7, 63.4 ± 25.4 ng/ml), Ch (112.1–1,526.1, 613 ± 295.1 ng/ml), Glu (778.2–5,998.6, 1,878.9 ± 914.8 ng/ml), _L_-Trp (11,006.5–44,853.4, 21,091.9 ± 5,915.5 ng/ml), Phe (5,734.4–16,919.3, 9,169.1 ± 1,703.7 ng/ml), and Tyr (10,098–32,219.6, 20,339.2 ± 4,809.8 ng/ml). The exposure levels of 5-HT, ACh, Glu, L-trp, and Phe reduced with aging, but the contents of 5-HIAA, Ch, and Tyr showed no significant tendency over time.

### Exposure Levels of Alkaloids in Different Physiological States of Mice

**FIGURE 5:**
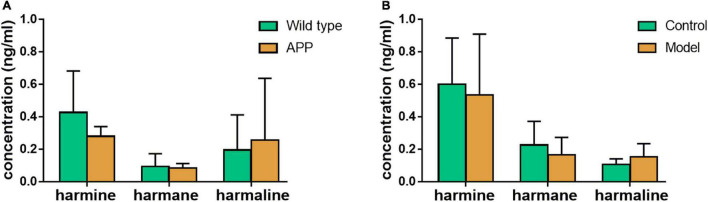
Exposure regulation of different healthy states. **(A)** wild-type mice compared with APP knock-in mice; **(B)** scopolamine-mode mice compared with normal mice.

As shown in Figure 5, in different physiological states of mice, the contents of harmine, harmane, and harmaline were different. As shown in Figure 5A, the contents of harmine and harmane in wild-type mice plasma (harmine: 0.43 ± 0.26 ng/ml, harmane: 0.095 ± 0.078 ng/ml) were higher than those in APP/PS1 double transgenic mice (harmine: 0.28 ± 0.060 ng/ml, harmane: 0.085 ± 0.028 ng/ml). However, the concentrations of harmaline in wide-type mice plasma (0.20 ± 0.22 ng/ml) were lower than those in APP/PS1 double transgenic mice (0.26 ± 0.38 ng/ml). As shown in Figure 5B, the contents of harmine and harmane in normal mice plasma (harmine: 0.60 ± 0.28 ng/ml, harmane: 0.23 ± 0.15 ng/ml) were higher than those in scopolamine-mode mice (harmine: 0.54 ± 0.37 ng/ml, harmane: 0.17 ± 0.11 ng/ml). By contrast, harmaline showed opposite results (control: 0.11 ± 0.034 ng/ml, model: 0.15 ± 0.080 ng/ml).

### Exposure Levels of Alkaloids in Different Developmental and Physiological States of Human

**FIGURE 6:**
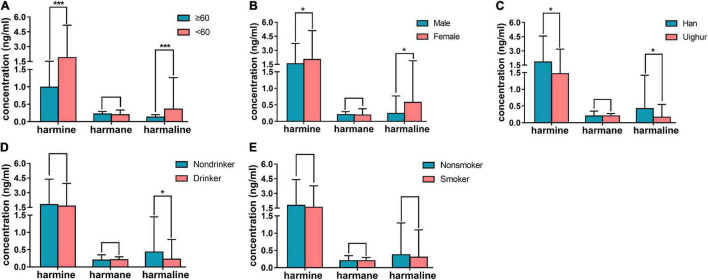
Results of human plasma determination. **(A)** contents of harmine, harmane, and harmaline in plasma of healthy individuals who were below 60 years old compared with those above 60 years old; **(B)** concentrations of the abovementioned alkaloids in plasma of healthy male individuals compared with female; **(C)** concentrations of the abovementioned alkaloids in plasma of Han compared with Uighur; **(D)** concentrations of the abovementioned alkaloids in plasma of healthy individuals who drink in daily life compared with those who were not; **(E)** concentrations of the abovementioned alkaloids in plasma of individuals who smoke in daily life compared with those who were not; Significant difference: *P < 0.05, ***P < 0.001.

In human plasma, the concentrations of nor-harmane were below the lower limit of detection, which could not be determined in most samples, and the results of the other three alkaloids are summarized in Figure 6. The content of harmine in plasma was higher than that of harmane and harmaline. The participants were divided into groups on the basis of age, gender, ethnicity, and living habit. Harmine (1.90 ± 3.27 ng/ml) and harmaline (0.36 ± 0.90 ng/ml) concentrations in individuals below 60 years old were significantly higher than those above 60 years old (harmine: 0.99 ± 0.54 ng/ml; harmaline: 0.13 ± 0.07 ng/ml; Figure 6A, *P* < 0.001). The plasma contents of harmine and harmaline in female and male were significantly different, among which the contents in female were higher (Figure 6B, *P* < 0.05). Detail information were shown as follows: harmine (female: 2.03 ± 3.07 ng/ml; male: 1.57 ± 2.16 ng/ml) and harmaline (female: 0.57 ± 1.32 ng/ml; male: 0.24 ± 0.53 ng/ml). In plasma samples obtained from participants with different ethnicities, the plasma concentrations of harmine and harmaline of Han nationality (harmine: 1.82 ± 2.74 ng/ml; harmaline: 0.42 ± 1 ng/ml) were higher than those of Uighur (harmine: 1.47 ± 1.72 ng/ml; harmaline: 0.17 ± 0.38 ng/ml), which showed significant difference (Figure 6C, *P* < 0.05). Drinking was a factor affecting the contents of harmaline, and the harmaline level of individuals who do not drink (0.42 ± 1.02 ng/ml) were higher than those who drink (0.22 ± 0.57 ng/ml; Figure 6D, *P* < 0.05). However, harmine was not affected (*P* > 0.05). Smoking did not affect the concentration of the three compounds in plasma (Figure 6E, *P* > 0.05). The concentrations of harmane showed no difference among different ages, genders, and ethnicities (*P* > 0.05).

## Discussion

The content of harmine, harmaline, harmane, and nor-harmane in different developmental and physiological states of rat was detected to explore the origin and function of such alkaloids. The concentrations of harmaline and harmane in some rat plasma samples were below the LLOQ, which were limited by the detecting condition. As for nor-harmane, this result was not determined in many samples; thus, nor-harmane had no results. Harmine was detected in newborn pup rat plasma and tissues. Harmine was discovered in each tissue of pup rats, including the brain, heart, liver, spleen, lungs, kidney, muscle, white fat, brown fat, and genital organ. In addition, researchers found harmine in pig and rat brain (Li et al., 2016). The exposure level of harmine in rat plasma decreased with age. Notably, harmine was found in the whole life of rat. In human plasma, harmine concentration changed with age, gender, race, and physiological and pathological status. Therefore, harmine is a naturally and widely distributed endogenous substance in mammals. The concentrations of harmaline and harmane were lower than that of harmine in most plasma of rats, mice, and human. Harmaline could turn to harmine *in vivo* with the presence of heme peroxidase (Wang et al., 2021), which might explain the contents of harmine were higher than that of harmaline under normal conditions. Based on the aforementioned research, harmine was more likely to be endogenous compounds than the other three alkaloids because harmine was detected in each rat, mouse, and human plasma, whereas the other three compounds were not.

Notably, harmine could be detected in plasma and other tissues of newborn rats without consuming any foodstuffs containing target alkaloids. Meanwhile, the fodder and bedding were free of harmine, and the exogenous disturbances from the growth environment of rats could be further excluded. Despite a weak signal response at the same peak time in harmine, the response was below the lower limit of detection. Thus, the trace amount of harmine, harmaline, harmane, and nor-harmane in fodder and bedding could be ignored. On the contrary, if the concentration of harmine was below the lower limit of detection, then the concentrations of harmine in fodder would reach 0.012 ng/g. The maximum daily food consumption of SD rats was approximately 20 g, and the bioavailability of harmine was 17% (Li et al., 2016; Wang et al., 2019). Considering that the blood volume accounted for 8% of the body weight of rats, combined with the daily intake of 20 g of each rat and the bioavailability of about 17% of harmine, the exogenous intake of harmine would only account for 1.3% of the total blood drug concentration. Therefore, based on the results of this experiment, harmine in newborn rat plasma and other tissues is natural rather than exogenous uptake.

Based on previous research of the synthesis of βCBs in plants, the P–S reaction was an essential part in the whole synthetic route, in which the strictosidine synthase was the vital enzyme in life (Stockigt et al., 2011; Zhu et al., 2015). The P–S reaction easily occurred without enzyme catalysis during cooking, including roasted coffee, barbecue, and toasted bread (Xie et al., 2021). The structure and function of strictosidine synthase in plants have been determined (Ma et al., 2006). Except for tryptamine and secologanin, other amines and aldehydes could be a substrate based on substrate specificity studies (Ma et al., 2006). Furthermore, a protein similar to strictosidine synthase was found in other life forms, including human (Cao and Wang, 2021). In addition, tryptamine, one of the substrates, is a popular compound in mammals (Abu Ghazaleh et al., 2015). Therefore, harmine may be synthesized *in vivo* in mammals by using tryptamine as a substrate under catalysis by synthesizing proteins similar to strictosidine in plants. Protein dysfunction may explain the decrease in harmine with aging. Age-dependent proteostasis decreases protein aberrant folding and aggregation, thereby leading to protein dysfunction (Hipp et al., 2014). Researchers found that the oxidative damage of membrane proteins and cytoplasmic proteins in the brain of an older person was higher than that of younger person (Granold et al., 2015). However, more experiments are needed to confirm the underlying mechanism.

The concentration of harmine in rats decreased with growth. Based on previous reports, harmine could stimulate proliferation of human neural progenitor cells and inhibit the dual specificity tyrosine-phosphorylation-regulated kinase, which regulated brain development (Dakic et al., 2016). Harmane could activate the firing and burst activity of dopamine neurons, and the increase in firing rate produced by harmane was greater than that produced by nicotine (Arib et al., 2010), which may imply that harmine has a similar effect because of the similar structure. In addition, tryptamine, the synthetic precursor of harmine, was widely distributed in the brain (Abu Ghazaleh et al., 2015). Therefore, harmine could play a role in the development of the nervous system. The sharp reduction of harmine in a developmental state may indicate that harmine may play an important role in primary functions in the early stage of nervous system growth and development.

The developmental and physiological states of newborn rats experienced great changes in a month, and pup rats from different maternal rats usually had inborn differences (Jungner et al., 2019). Thus, the sampling time and grouping are important factors. The dates of each sampling point were obtained from 10 different maternal rats to avoid inborn differences and make the experiment more accurate. The sampling time was different because the samples were not delivered on the same day. Thus, the experiment eliminated congenital difference of pup rats and reflected the variation trend of harmine in various tissues and plasma with the development of pup rats.

The concentration of harmine in rat plasma decreased with age. The brain exhibited signs of compromised bioenergetics with aging, including inflammation, accrual of oxidatively modified molecules, and other impairments. In addition, aging individuals were vulnerable to AD or other neurodegenerative diseases (Corriveau et al., 2017). Alzheimer’s disease (AD) was a deadly and progressive neurodegenerative disorder, and the pathogenesis of the disease had no clear conclusion at present (Hampel et al., 2018). Researchers directly associated the disease with aging but not specifically with the theories of aging in general. Distinguishing normal aging from AD is difficult because aging is a main risk factor for acquiring AD (Hargis and Blalock, 2017). Researchers showed the relationship between aging and AD, and they regarded the aging rodent models as an AD model (Hargis and Blalock, 2017). Many factors of aging could alleviate AD phenotypes, and drugs and treatment for AD could slow the aging phenotypes (Xia et al., 2018). Harmine is a compound that enhanced the spatial cognition of AD mouse models and regulated the concentration of various neurotransmitters (Li et al., 2018). Based on the pharmacological effects of harmine on AD treatment, the lack of harmine might cause or accelerate the development of AD during aging. Moreover, harmine is the inhibitor of dual-specificity tyrosine phosphorylation-regulated kinase 1A (DYRK1A) (Sitz et al., 2008). The expression of DYRK1A increases with age, whereas DYRK1A could promote the formation of characteristic pathological hallmarks of AD by direct phosphorylation of tau and Aβ (Sitz et al., 2008; Chaves et al., 2020).

The contents of 5-HT, ACh, Glu, _L_-Trp, and Phe in rats reduced with aging. In addition, the contents of such neurotransmitters were lower in plasma of AD mouse models with intraperitoneal injection of scopolamine than those of normal mice (Li et al., 2018). The AD was associated with inadequate levels of a variety of neurotransmitters (Kumar et al., 2015). Neurotransmitters played a significant role in brain circuit involved in many aspects of learning and memory, particularly serotonergic, glutamatergic, and cholinergic neurotransmitters (Kumar et al., 2015). This result indicated the relationship between aging and AD.

The content of harmine, harmaline, and harmane in two mouse models, including APP/PS1 double transgenic mice and scopolamine-induced memory impairment model mice, showed no significant difference compared with the control. The AD model mice were determined to clarify the relationship between AD and alkaloids. Scopolamine was a non-selective antagonist of the muscarinic cholinergic receptor, which led to cognitive deficits associated with the reduction of cholinergic neurotransmission (Tang, 2019). The APP/PS1 double transgenic mice showed typical Aβ pathology and memory impairment in an age-dependent manner (Esquerda-Canals et al., 2017). Although harmine, harmaline, and harmane could enhance the cognitive ability of AD mouse models, the occurrence of disease played a little role in the regulation of these alkaloids.

Harmaline and harmine showed a significant difference among young and old people. Based on previous statistics, old people may suffer from AD (Association, 2016). The reduction of harmine and harmaline may account for the easy attack because of an efficient pharmacology in AD (Li et al., 2017a). In addition, when the neuronal cell of the central nervous system was senescent, aged neurons showed signs of impaired cellular signaling, which exhibited a memory decline (Swenson et al., 2019). The results were consistent with the reduction of harmine in an aging rat plasma.

Based on the results, the contents of harmaline and harmine in female plasma were higher than those of male plasma (*P* < 0.05). The result may be due to the different expression levels of cytochrome enzyme in women and men. Harmaline and harmine were metabolized by CYP2D6 (Li et al., 2016). The expression level of CYP2D6 in female was lower than that of male based on research, and the response to opioids showed gender differences (Bebia et al., 2004; Lopes et al., 2020). The difference in the expression of CYP2D6 accounted for the significant difference in the plasma contents of harmine and harmaline in female and male. On the contrary, no gender difference was associated with CYP1A2 activity (Bebia et al., 2004). Thus, no difference in gender was found among the plasma concentrations of harmane.

The concentrations of harmaline and harmine in plasma of Han subjects were higher than those of Uyghur (*P* < 0.05). The CYP2D6 was the main metabolic enzyme of harmaline and harmine (Li et al., 2017b). A reduced functional allele CYP2D6 * 10 was important in Asian population, and Asians have a high frequency of reduced functional allele (median = 41%), CYP2D6 * 10 contributed to the population shift to the right of the metabolic rates indicating slower metabolism (Bradford, 2002). Researchers compared the expression of CYP2D6 * 10 in the Han and Uyghur blood. The result showed that the expression of CYP2D6 * 10 in Han was higher than that in Uyghur’s (Zuo et al., 2012). Consequently, the metabolic rate of CYP2D6 in Uyghur was faster than that in Han’s, and the concentrations of harmaline and harmine in Han plasma were higher than those of Uyghur. Different from harmine and harmaline, CYP1A2 was the main metabolic enzyme of harmane (Herraiz et al., 2008). Moreover, no significant difference of CYP1A2 was found between Han and Uyghur. Therefore, the plasma concentrations of harmane showed no difference between Han and Uyghur.

No significant differences in such alkaloids were found among smokers and drinkers, except for harmaline. The plasma concentration of harmaline in drinkers was lower than that of non-drinker (*P* < 0.05). This finding was consistent with various reports, that is, ethanol consumption could adversely affect AD and increase the risk of AD development (Huang et al., 2018). Increasing consumption of hard liquor could increase the rate of cognitive decline compared with mild to moderate drinks (Rehm et al., 2019). Such studies showed the correlation between drinking and disease, but no finding indicated a causal impact of alcohol on AD. Thus, the degree of drinking and standardization of alcohol may be a vital element for further research.

## Conclusion

Harmine is a widespread βCBs in mammals, and it could be discovered in the plasma of rat, mouse, and human. Harmine was found in newborn rat plasma and many tissues without any consumption. In addition, harmine showed a high correlation with growth (aging), gender, race, and physiological status. These results revealed that harmine might be a potential biomarker in diagnosis of AD and effective remedy of AD. However, further studies were warranted to determine the underlying mechanism of endogenous synthesis and the specific function of harmine in mammals. This study was the first to investigate the variation of harmine concentration in mammals in different developmental and health states. This study laid the foundation for the discovery of novel AD biomarkers and provided insights into the development of functional foods and drugs.

## Data Availability Statement

The original contributions presented in the study are included in the article/Supplementary Material, further inquiries can be directed to the corresponding author/s.

## Ethics Statement

The studies involving human participants were reviewed and approved by the Research Ethics Committee of Kashi Prefecture First People’s Hospital, and all participants provided informed consent (No. 2017-03; Approval date: February 28, 2017). The patients/participants provided their written informed consent to participate in this study. The animal study was reviewed and approved by the Animal Ethics Committee of Shanghai University of Traditional Chinese Medicine (No. PZSHUTCM190912018; Approval date: 12 September, 2019).

## Author Contributions

NC performed most of the experiments, analyzed the data, and wrote the manuscript. SL detected the concentrations of harmine, harmaline, and harmane in plasma of AD mode mice and control. AX and XZ collected plasma and basic information of healthy volunteers and AD patients. ZK provided the idea of the experiment about pup rats. ML helped to conduct the experiment. GD collected plasma samples of aging rats. XC helped to develop the UPLC-MS/MS detection. CW proposed the project and gave suggestions on the revision. All authors contributed to the article and approved the submitted version.

## Conflict of Interest

The authors declare that the research was conducted in the absence of any commercial or financial relationships that could be construed as a potential conflict of interest.

## Publisher’s Note

All claims expressed in this article are solely those of the authors and do not necessarily represent those of their affiliated organizations, or those of the publisher, the editors and the reviewers. Any product that may be evaluated in this article, or claim that may be made by its manufacturer, is not guaranteed or endorsed by the publisher.

## Abbreviations

β CBs: β-Carboline alkaloids
5-HT: 5-Hydroxytryptamine
5-HIAA: 5-Hydroxyindole-3-acetic acid
ACh: Acetylcholine chloride
AChE: Acetylcholinesterase
AD: Alzheimer’s disease
Ch: Choline chloride
_L_-Glu: _L_-Glutamic acid
_L_-Phe: _L_-Phenylalanine
_L_-Trp: _L_-Tryptophan
_L_-Tyr: _L_-Tyrosine
MAO-A: Monoamine oxidase A
P–S reaction: Pictet–Spengler reaction.
