# Supplementary material for: Dynamic Changes of Endogenic or Exogenic β-Carboline Alkaloid Harmine in Different Mammals and Human in vivo at Developmental and Physiological States
Source: Front Aging Neurosci. 2022 Jan 14;13:773638. doi: 10.3389/fnagi.2021.773638 (PMC8794950; doi:10.3389/fnagi.2021.773638)
Supplement: Supplementary file 1 [file Data_Sheet_1.pdf]

Supplementary materials

Dynamic Changes of Endogenic or Exogenic β -Carboline Alkaloid Harmine in Different Mammals and Human in vivo at Developmental and Physiological States

Contents of Figures and Tables

Table S1. The optimized MS analytical parameters of alkaloids and IS

Table S2. Accuracy, precision, extraction recovery and matrix effect of harmaline, harmine, harmane and nor-harmane in plasma

Table S3. Stability of harmaline, harmine, harmane and nor-harmane in plasma

Table S4. Sampling time of pup rats and their gender. Every sampling time contain 10 rats (half male and half female) at least. (One pup rat of NO.5 rat was dead. /: no sampling)

Table S5. The analyte peak area of harmine, harmaline, harmane and nor-harmane in fodder and bedding

Fig. S1. The chromatograms of harmine, harmaline, harmane and nor-harmane

Fig. S2. The amounts of harmine in various tissues with the growth process of pup rats. The line shows amount of harmine in various tissues; the column shows weight of pup rats

Table S1. The optimized MS analytical parameters of alkaloids and IS.

ID	Q1（Da）	Q3（Da）	DP（volts）	EP（volts）	CE（volts）	CX（volts）
Harmaline	215.100	172.000	18.000	7.000	42.000	22.000
Harmine	213.100	170.200	50.000	8.000	41.000	11.000
Harmane	183.100	115.200	100.000	5.000	44.000	10.000
Nor-harmane	169.000	115.100	135.000	10.000	46.000	16.000
IS (tacrine)	199.100	144.100	135.000	5.000	50.000	17.000

Table S2. Accuracy, precision, extraction recovery and matrix effect of harmaline, harmine, harmane and nor-harmane in plasma.

	Concentration (ng/ml)	Accuracy(%, mean±SD)	Precision(%, RSD)	Extraction recovery(%, mean±SD)	Matrix effect (%, mean±SD)
harmaline	0.05	96.55±3.06	4.77	119.83±6.11	118.52±1.15
	0.2	99.77±2.71	4.01	106.10±5.53	107.53±3.09
	5	104.60±4.03	2.74	88.07±2.86	85.76±0.78
	20	90.25±2.82	3.72	94.36±3.52	94.24±3.55
harmine	0.05	94.17±3.02	1.98	100.78±6.72	101.21±7.99
	0.2	104.62±4.15	3.85	86.52±2.72	88.42±4.52
	5	103.42±4.88	3.38	90.63±7.84	88.11±3.97
	20	85.48±2.75	4.53	98.85±2.05	96.61±6.47
harmane	0.05	97.65±2.62	3.18	94.23±5.52	93.78±7.69
	0.2	100.57±2.46	5.61	96.32±4.32	94.17±5.42
	5	103.40±4.94	3.70	89.21±3.51	89.21±3.51
	20	88.17±3.14	4.55	97.37±6.13	96.27±1.50
nor-harmane	0.05	99.30±2.72	9.03	98.24±12.58	104.35±6.68
	0.2	101.52±5.39	7.89	97.17±6.57	93.37±4.71
	5	104.55±5.34	5.78	84.52±1.07	90.05±1.74
	20	86.65±2.78	5.18	87.17±6.83	84.57±2.29

Table S3. Stability of harmaline, harmine, harmane and nor-harmane in plasma.

	Concentration (ng/ml)	RT for 4h RSI (%)	Frozen for 16 months RSD (%)	Freeze-thaw RSD (%)	Prepared sample RSD (%)
harmaline	0.05	9.10	8.97	3.93	1.31
	0.2	3.20	2.17	4.29	4.30
	5	6.20	4.85	4.22	0.62
	20	3.74	4.13	3.62	2.95
harmine	0.05	2.19	7.75	5.33	2.65
	0.2	4.42	9.86	7.96	4.24
	5	1.74	3.83	2.63	3.32
	20	1.56	3.47	3.59	3.50
harmane	0.05	3.18	2.95	3.95	3.43
	0.2	2.39	1.09	3.20	5.00
	5	2.22	2.74	4.90	0.30
	20	1.19	2.14	6.51	1.79
nor-harmane	0.05	1.36	8.92	7.65	0.49
	0.2	4.00	6.69	7.57	1.80
	5	5.35	5.54	6.71	1.08
	20	3.52	3.01	5.65	1.97

Table S4. The sampling time of pup rats and their gender. Every sampling time contain 10 rats (half male and half female) at least.(One pup rat of NO.5 rat was dead. /: no sampling)

Number	Litter size	Delivery time	Sampling time (month day)/sex										
			1	3	5	7	9	12	15	18	21	25	29
1	13	2019.9.21	9.21♂	9.23♀	9.25♀	9.27♂	9.29♂	10.2♂	10.5♀	10.8♀	10.11♀	10.15♀	10.19♂
2	10	2019.9.24	9.24♂	9.26♂	9.28♀	9.30♀	10.2♀	10.5♀	10.8♂	10.11♀	10.14♂	10.18♀	/
3	13	2019.9.21	9.21♂	9.23♀	9.25♀	9.27♂	9.29♀	10.2♂	10.5♂	10.8♀	10.11♂	10.15♀	10.19♀
4	11	2019.9.25	9.25♀	9.27♀	9.29♂	10.1♂	10.3♂	10.6♂	10.9♀	10.12♂	10.15♀	10.19♀	10.23♂
5	10	2019.9.21	9.21♀	9.23♂	9.25♂	9.27♀	9.29♀	10.2♂	10.5♂	10.8♂	/	/	10.19♀
6	9	2019.9.26	9.26♂	9.28♀	9.30♀	10.2♂	10.4♂	10.7♀	/	/	10.16♀	10.20♂	10.24♀
7	11	2019.9.27	9.27♀	9.29♂	10.1♂	10.3♀	/	10.8♀	10.11♀	10.14♂	10.17♂	10.21♂	10.25♂
8	10	2019.9.26	9.26♀	9.28♀	9.30♂	/	10.4♂	10.7♀	10.10♂	10.13♀	10.16♀	10.20♂	10.24♀
9	10	2019.9.26	9.26♀	9.28♂	9.30♀	10.2♂	10.4♀	/	10.10♂	10.13♂	10.16♂	10.20♂	10.24♂
10	11	2019.9.28	9.28♂	9.30♀	10.2♂	10.4♀	10.6♀	10.9♂	10.12♀	10.15♀	10.18♀	10.22♀	10.26♀
11	11	2019.9.28	9.28♀	9.30♂	10.2♀	10.4♀	10.6♂	10.9♀	10.12♀	10.15♂	10.18♂	10.22♂	10.26♂

Table S5. The analyte peak area of harmine, harmaline, harmane and nor-harmane in fodder and bedding.

Name	Fodder/ analyte peak area	Bedding/ Analyte peak area	LLOQ peak area
Harmine	1.11e+004	5.33e+004	1.77e+005
Harmaline	2.08e+004	3.15e+004	7.77e+004
Harmane	2.70e+004	2.58e+004	4.87e+004
Nor-harmane	2.99e+004	4.58e+004	7.35e+004

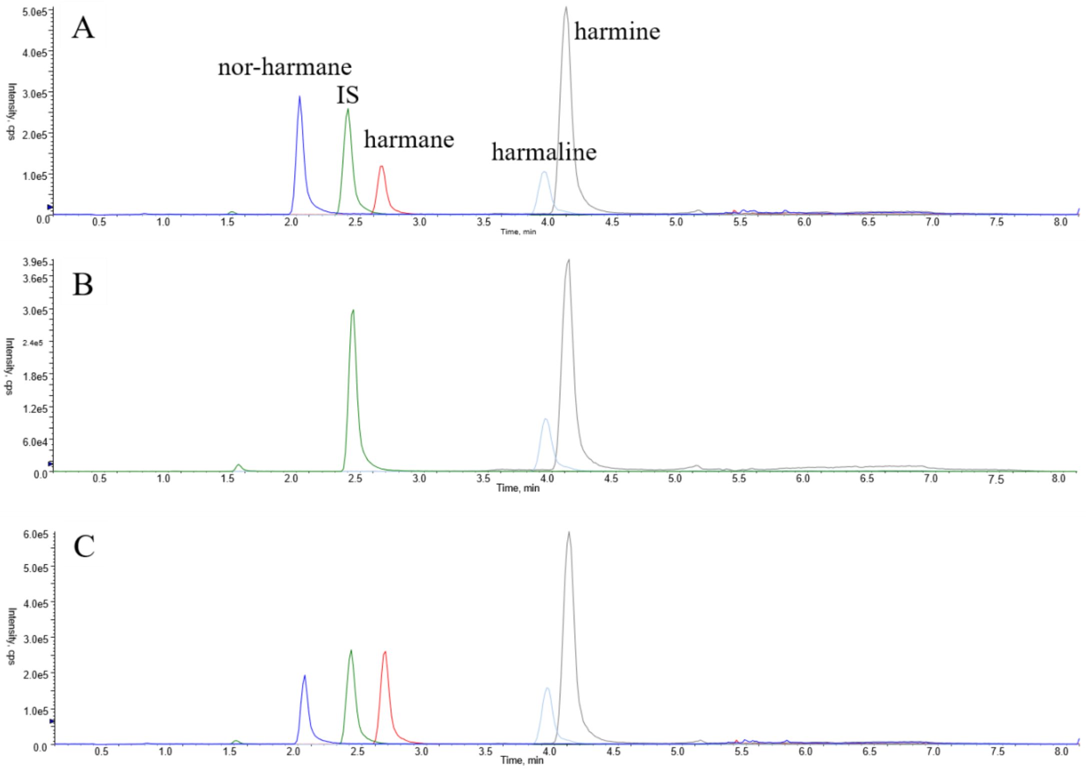

Fig. S1. The chromatograms of harmine, harmaline, harmane and nor-harmane. A: Blank plasma spiked with various alkaloids at LLOQ and IS;B: Representative MRM chromatograms of blank plasma sample; C: Plasma sample. The blue line represents the chromatogram of nor-harmane; the green line represents harmane; the red line represents IS; the wathet line represents harmaline; the gray line represents harmine.

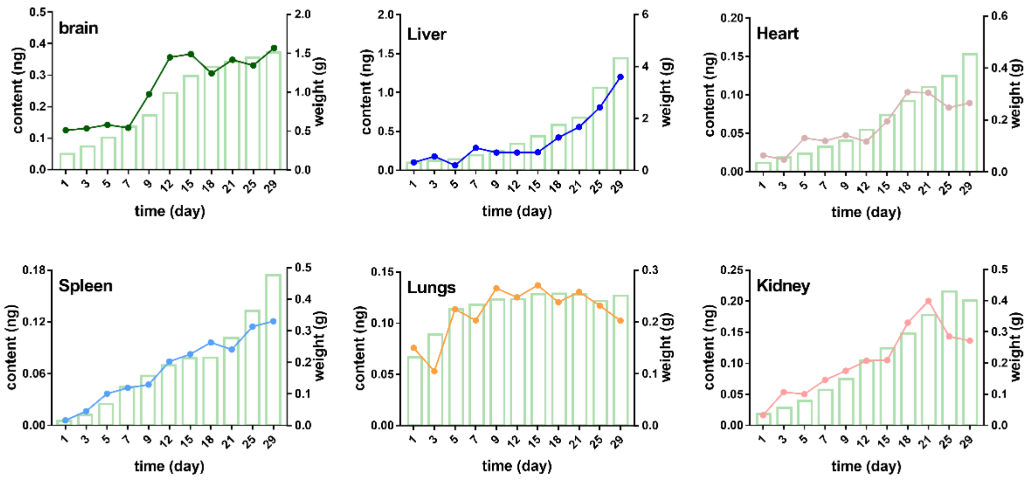

Fig. S2. The amounts of harmine in various tissues with the growth process of pup rats. The line shows amount of harmine in various tissues; the column shows weight of pup rats.
